# Machine learning for prediction of bronchopulmonary dysplasia-free survival among very preterm infants

**DOI:** 10.1186/s12887-022-03602-w

**Published:** 2022-09-13

**Authors:** Rebekah M. Leigh, Andrew Pham, Srinandini S. Rao, Farha M. Vora, Gina Hou, Chelsea Kent, Abigail Rodriguez, Arvind Narang, John B. C. Tan, Fu-Sheng Chou

**Affiliations:** 1 Loma Linda University School of Medicine, Loma Linda, CA USA; 2 Division of Neonatology, Department of Pediatrics, Loma Linda University School of Medicine, Loma Linda, CA USA; 3 Business Intelligence and Data Governance, Loma Linda University Health, Loma Linda, CA USA; 4 Huckleberry Labs, Inc., Irvine, CA USA; 5 Kaiser Permanente Riverside Medical Center, 10800 Magnolia Ave., Riverside, CA 92505 USA

**Keywords:** Bronchopulmonary dysplasia, Machine learning, Predictive modeling, Preterm infants

## Abstract

**Background:**

Bronchopulmonary dysplasia (BPD) is one of the most common and serious sequelae of prematurity. Prompt diagnosis using prediction tools is crucial for early intervention and prevention of further adverse effects. This study aims to develop a BPD-free survival prediction tool based on the concept of the developmental origin of BPD with machine learning.

**Methods:**

Datasets comprising perinatal factors and early postnatal respiratory support were used for initial model development, followed by combining the two models into a final ensemble model using logistic regression. Simulation of clinical scenarios was performed.

**Results:**

Data from 689 infants were included in the study. We randomly selected data from 80% of infants for model development and used the remaining 20% for validation. The performance of the final model was assessed by receiver operating characteristics which showed 0.921 (95% CI: 0.899–0.943) and 0.899 (95% CI: 0.848–0.949) for the training and the validation datasets, respectively. Simulation data suggests that extubating to CPAP is superior to NIPPV in BPD-free survival. Additionally, successful extubation may be defined as no reintubation for 9 days following initial extubation.

**Conclusions:**

Machine learning-based BPD prediction based on perinatal features and respiratory data may have clinical applicability to promote early targeted intervention in high-risk infants.

## Background

Bronchopulmonary dysplasia (BPD) was first described by Northway et al. in 1967 as a new lung disease of preterm infants following respiratory distress syndrome [1]. Over the past 50 years since its first characterization, medical technology and clinical management have evolved. Accordingly, the pathology of BPD evolved from primarily necrotic bronchiolitis and fibrotic changes in the lung tissues to alveolar simplification in the post-surfactant era [1, 2]. BPD is associated with long-term cardiopulmonary complications as well as neurodevelopmental disadvantages, including cerebral palsy, vision and hearing deficits, mental and psychomotor impairments [3–9].

Due to how its defined clinically, the diagnosis of BPD is rather subjective. Most contemporary practices follow the 2001 NICHD Workshop definition to diagnose BPD at 36 weeks postmenstrual age (PMA) [10]. Severity stratification was based on the duration of supplemental oxygen and respiratory support use. A revised operational definition was proposed after the 2016 Workshop [11]. In 2019, a systematic approach correlating 18 operational definitions of BPD with toddler-age respiratory and neurodevelopmental outcomes suggested that the best way to diagnose and grade BPD was by respiratory support mode at 36 weeks PMA. Notably, the extent of supplemental oxygen use was not needed [12].

To our knowledge, the concept of a developmental origin of BPD was first explicitly mentioned by Thébaud et al. recently in their extensive review article on BPD [13]. Indeed, gestational age (GA) is the best predictor of BPD [14]. Additionally, genetic factors such as race/ethnicity and sex also play a role [15–18]. Moreover, modifiable perinatal factors such as maternal smoking, chorioamnionitis, placental insufficiency, as well as volutrauma, barotrauma, oxygen toxicity, and inflammation from prolonged mechanical ventilation use have been associated with BPD [19–23]. The modifiable factors may reflect on the trajectory of respiratory support in the early postnatal period. As a result, distinct patterns of lung disease based on supplemental oxygen use have been characterized as early as the first two weeks of life [24, 25]. These early predictors led to the development of the BPD outcome estimator using 4 demographic and 2 respiratory factors which allowed risk stratification at six different days of life in the first 4 weeks of life [26, 27]. After external validation has been performed, the predictive power was found to be lacking, with the area under the receiver’s operating characteristic curves ranging only between 0.73–0.76 [26, 28, 29]. We and others also did not find it particularly useful clinically [30, 31]. Center effect likely plays an impactful role, and a cutoff probability level needs to be individualized for each unit [29, 30, 32].

In recent years, machine learning algorithms have become more accessible to clinical researchers. Machine learning algorithms are able to detect patterns in data that are invisible to the human eye, and it can be used as a more appropriate data analysis strategy for multifactorial pathologies such as BPD. In this project, we sought to investigate whether perinatal features and the trajectory of early-life respiratory support can be used to predict BPD-free survival using a machine learning algorithm.

## Methods

### Study participants

Machine learning-based predictive modeling analyzed a retrospective dataset encompassing infants born between 2013 to 2020 with a birth gestation of 30 weeks 3 days or less admitted to the neonatal intensive care unit (NICU) at the Loma Linda University Children’s Hospital (LLUCH) or at Riverside University Health System (RUHS) [33]. The study was approved by the Institutional Review Boards (LLUCH IRB#: 520338; RUHS IRB#: 1689889) of both institutes with waiver of informed consent due to the retrospective nature of the study. Infants without complete data for perinatal and respiratory features, those that died in the first 28 days of life (DOL), or those that were transferred out of NICU before an assessment for a BPD diagnosis could be made, were excluded. Infants with gestation longer than 30 weeks 3 days were not included because their risk for BPD were low.

### Feature engineering and data extraction

We followed the study by Morrow et al. on the risk analysis of perinatal factors in association with BPD development to design perinatal features [18], which included assigned sex (female or male), self-reported race/ethnicity (White, Black, Hispanic, Asian, or other), birth gestation, birth weight category, and maternal smoking during pregnancy. For birth gestation, instead of categorizing based on complete weeks, we used the closest gestation in full week for assignment. For example, an infant born at gestational age 29 weeks 3 days would be assigned to the 29-week category, whereas an infant born at gestational age 29 weeks 4 days would be assigned to the 30-week category. For birth weight category assignment, we obtained sex- and gestational age-specific birth weight percentile using the 2013 Fenton growth charts [34]. Infants were then categorized into small (< 10^th^ percentile), appropriate (10^th^-90^th^ percentile), or large (> 90^th^ percentile) for gestational age.

For respiratory features, we only included the respiratory support modes during model development. Our reasoning was that the respiratory support mode should reflect the severity, reversibility of illness and respiratory maturity levels, noting that it is the only variable strictly based on the current physician’s orders. The respiratory support modes were classified into five categories: (1) high-frequency ventilation (HFV, including high-frequency jet ventilator or high-frequency oscillator), (2) conventional mechanical ventilation (CMV, including pressure-control mode, volume-control mode, or invasive neurally-adjusted ventilatory assist (NAVA)), (3) non-invasive mechanical ventilation (NIMV, defined as non-invasive ventilatory mode which provides a peak inspiratory pressure, including non-invasive positive pressure ventilation (NIPPV) or non-invasive NAVA), (4) continuous positive airway pressure (CPAP) or high-flow nasal cannula (HFNC), and (5) low-flow nasal cannula (LFNC) or no support. Notably, the reason we grouped CPAP and HFNC together was that some providers in our group had a preference of using HFNC at 8–10 L per minute to “mimic” CPAP.

Perinatal data were extracted from the backend database of the electronic medical records (A.N.). Respiratory mode data were available in the daily flowsheets documented by the respiratory therapists. These data were first extracted from the backend database (A.N.), followed by manual curation using a custom web app designed specifically for the purpose. Respiratory mode data were collected based on the respiratory mode the infants were receiving at the end of each 24-h interval after birth for 14 consecutive intervals (instead of by calendar dates). Each DOL indicates a 24-h interval, or a complete day, throughout the manuscript.

### Outcome definition

BPD-free survival outcome was defined as survival until at least 36 weeks PMA and no respiratory support needs at 36 weeks PMA. Notably, BPD was defined based on the 2019 Jensen criteria, meaning that respiratory support in the first 28 days of life was not taken into consideration for BPD diagnosis [12].

### Model training and validation

Supervised machine learning based on the defined outcome as described above using a random forest algorithm was performed on a randomly selected 80% of the complete dataset. The remaining 20% of the data was used for internal validation. A random forest algorithm, a decision tree-based algorithm, was the algorithm of choice for this study partly because all features were categorical in nature. Four random-forest models each with 500 trees planted and a tenfold cross-validation repeated 10 times were trained using the *caret (6.0–89)* and *ranger (0.13.1)*packages for R [35–37].

- *Model 1: perinatal features only*
- *Model 2: Respiratory data from DOL1*
- *Model 3: Respiratory data from DOL1-7*
- *Model 4: Respiratory data from DOL1-14*

The probabilities of outcome prediction from the above four random-forest models were subsequently used to develop additional ensemble models by using a generalized logistic regression algorithm:

- *Model 5: Model 1 and Model 2*
- *Model 6: Model 1 and Model 3*
- *Model 7: Model 1 and Model 4*

An interaction term to assess the interaction of probabilities from the two random-forest models was introduced in generalized logistic regression modeling.

Model performance was assessed by the receiver operating characteristic area under the curve (ROC AUC) as well as by overall accuracy, positive predictive value, and negative predictive value. Youden’s J statistics was utilized to calculate the optimal cutoff threshold for binary outcome prediction (Yes or No for BPD-free survival) which was needed in order to obtain the latter performance parameters. The *pROC*(1.18.0) package for R was used for these analyses [38]. During the validation process using the testing dataset, the same cutoff threshold generated from applying the training dataset to the model was applied for each corresponding model.

### Simulation

Three scenarios were designed for simulation. The goal for the first simulation scenario was to validate the model. The goal for the second and the third simulation scenarios assessed whether the model could be used to answer common clinical questions about BPD.

1. *Scenario 1*
  In this scenario, we explored if the projection of BPD-free survival prediction increased with increasing birth gestation and decreased with longer intubation time following birth. To test, we created three sets of simulated patients, each set born at a different gestational age (23, 26, and 29 weeks). All infants were intubated within DOL1 and placed on HFV. Each set contained 5 patients, all extubated at a different timepoint (after 1, 3, 6, and 10 complete days, or remained intubated for all 14 days). All the simulated infants were non-Hispanic White, female, appropriate for gestational age, and without *in utero* exposure to smoking.
2. *Scenario 2*
  In this scenario, there were two sets of appropriate-for-gestational age non-Hispanic White female infants born at 26 weeks’ gestation without antenatal smoking exposure. The infants were all intubated within the first 24 hrs of life and placed on HFV. In each set, infants were extubated at different time points (after DOL 1, 3, 6, or 10) to either NIMV or CPAP/HFNC. This scenario was used to assess the differences in BPD-free survival between the two non-invasive modes. Of note, the scenario was not designed to compare superiority between NIMV vs. CPAP/HFNC, and should not be confused with clinical trials.
3. *Scenario 3*
  In this scenario, a group of appropriate-for-gestational age, non-Hispanic White, female infants without antenatal smoking exposure were born at 26 weeks’ gestation. The infants were intubated by the end of DOL1, subsequently extubated to CPAP by the end of DOL2, followed by reintubation after various periods of time ranging from 1 to 12 complete days of extubation. After reintubation, the infant was placed on CMV. The control infant did not require reintubation. This scenario assessed the duration of time needed for the infant to remain extubated in order to have comparable BPD-free survival as compared to the control infant.

### Statistical analysis

Descriptive statistics were performed for demographic comparison. χ^2^ tests were used for categorical variables. Student’s *t*-tests or Mann–Whitney U tests were employed for continuous variables. *P*-value < 0.05 was considered statistically significant.

BPD-free survival prediction probabilities were compared by supplying probability as mean and standard error multiplied by the square root of total trees planted (*n* = 500) as standard deviation using a Welch-modified two-sample *t*-test assuming unequal variance. *P*-value < 0.05 was considered as statistically significant.

All analyses were performed at Loma Linda University. Only IRB approved study personnel had access to private health information.

## Results

**Fig. 1:**
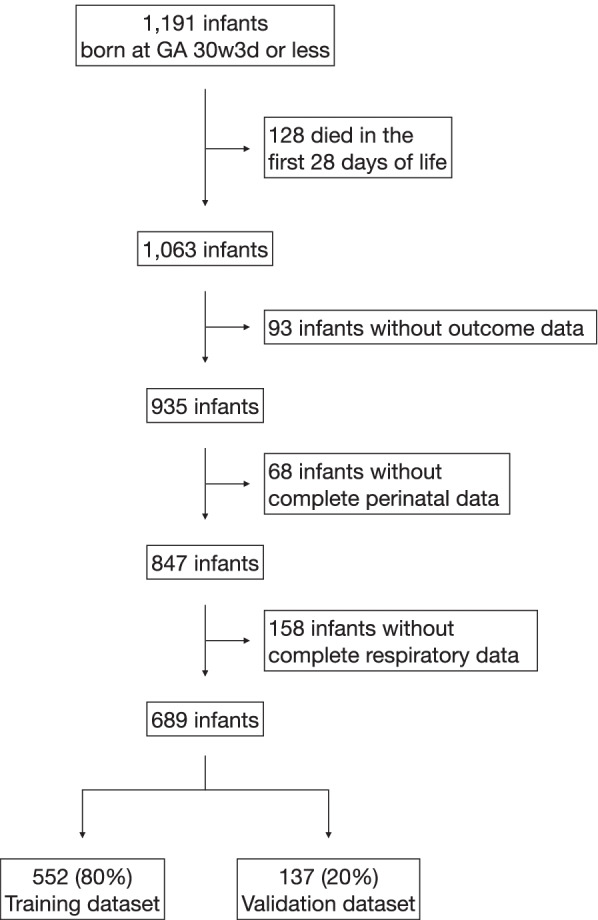
A flow chart depicting the selection of study participants

Out of a total of 1,191 infants who met the gestational age criteria, 128 died before 28 days of life and were excluded. Among the remaining 1,063 infants, outcome data were available for 935 infants, complete data for perinatal features were available in 847 infants, and respiratory data were available in 689 infants. Perinatal and respiratory data from these infants were used in the study (Fig. 1). Data from randomly selected 552 (80%) infants were used for model training, and data from the remaining 137 (20%) infants were used for validation (Fig. 1). Demographic information and the number of infants with each respiratory support mode at various DOL are summarized in Tables 1 and 2, respectively.

**Table 1:** Demographic characteristics of the study participants

	All Infants (*n* = 689)	Training Dataset (*n* = 552)	Validation Dataset (*n* = 137)	*P*-value ^*^
Birth gestation in week (mean ± SD)	27.6 ± 2.1	27.6 ± 2.0	27.5 ± 2.1	0.832
Birth weight in gram (mean ± SD)	1,034 ± 334	1,033 ± 333	1,042 ± 352	0.814
Birth weight z-score (mean ± SD)	0.09 ± 0.99	0.07 ± 1.00	0.16 ± 0.97	0.888
Small for gestational age, n (%)	58 (8)	51 (9)	7 (5)	0.765
Female, n (%)	346 (50)	272 (49)	74 (54)	0.107
Maternal smoking, n (%)	64 (9)	52 (9)	12 (9)	0.771
Surfactant within 24 h of life, n (%)	348 (51)	272 (49)	76 (55)	0.216
Race/ethnicity, n (%)				0.161
*White*	158 (23)	129 (23)	29 (21)	
*Black*	107 (16)	83 (15)	24 (18)	
*Hispanic*	363 (53)	291 (53)	72 (53)	
*Asian*	36 (5)	25 (5)	11 (8)	
*Other*	25 (4)	24 (4)	1 (1)	
No BPD	352 (51)	282 (51)	70 (51)	0.335
BPD	337 (49)	270 (49)	67 (49)	0.120
*Grade 1*	176 (26)	145 (26)	31 (23)	
*Grade 2*	133 (19)	102 (19)	31 (23)	
*Grade 3 or Died*	28 (4)	23 (4)	5 (3)	

^*^Comparison between the training and the testing datasets

**Table 2:** A table depicting the number of infants on each indicated respiratory support mode on Day of life 1, 7, and 14

Day	Respiratory support	Training Dataset		Testing Dataset	
		No BPD (*n* = 282)	BPD (*n* = 270)	No BPD (*n* = 70)	BPD (*n* = 67)
1	HFV	12	35	3	9
	CMV	51	132	15	39
	NIMV	53	40	16	9
	CPAP/HFNC	155	62	34	10
	LFNC/RA	11	1	2	0
7	HFV	6	57	2	10
	CMV	19	83	5	26
	NIMV	16	54	7	18
	CPAP/HFNC	203	75	45	13
	LFNC/RA	38	1	11	0
14	HFV	3	58	1	12
	CMV	12	83	2	26
	NIMV	23	52	8	15
	CPAP/HFNC	165	73	35	14
	LFNC/RA	79	4	24	0

*HFV* high-frequency ventilation, *CMV* conventional mechanical ventilation, *NIMV* non-invasive mechanical ventilation, *CPAP* continuous positive airway pressure, *HFNC* high-flow nasal cannula, *LFNC* low-flow nasal cannula, *RA* room air

The performances of the random forest and the ensemble models were listed in Table 3. Model 1 (perinatal features only) had a ROC AUC of 0.861 with the training dataset and 0.786 with the testing dataset. Using the probability cutoff threshold calculated for binary outcome prediction based on the training dataset (55%), we found positive and negative predictive values to be 0.773 and 0.802, respectively, upon model validation with the testing dataset. The ROC AUC increased with more respiratory data available for training, from 0.724 with only 1 day of data to 0.900 with all 14 days of data. The ensemble model combining perinatal features and all 14 days of respiratory data (Model 7) provided the best prediction with ROC AUC of 0.921 in the training dataset, and 0.899 in the testing dataset. Using a cutoff threshold of 48.8% for binary outcome prediction, the overall predicting accuracy was 85.1% and 81.0% in the training and the testing dataset, respectively. In the testing dataset, the positive predictive value was 0.855 and the negative predictive value was 0.773.

**Table 3:** A table detailing performance measures for various random forest models predicting bronchopulmonary dysplasia-free survival. The training dataset was used for model development. The testing dataset was used for model validation

Model ID	Data included in model training	ROC AUC (95% CI)	Cutoff threshold	Youden’s J value	Accuracy (95% CI)	PPV	NPV
**Training Dataset**							
1	Perinatal features only	**0.861 (0.831–0.891)**	0.550	0.571	0.786 (0.750–0.820)	0.773	0.802
2	DOL1 respiratory feature	0.724 (0.684–0.764)	0.537	0.395	0.699 (0.659–0.737)	0.680	0.726
3	DOL1-7 respiratory features	0.866 (0.836–0.896)	0.341	0.618	0.808 (0.773–0.840)	0.849	0.773
4	DOL1-14 respiratory features	0.900 (0.875–0.926)	0.510	0.676	0.839 (0.805–0.869)	0.816	0.866
5	Ensemble of Models 1 & 2	0.875 (0.847–0.904)	0.521	0.624	0.812 (0.776–0.843)	0.827	0.796
6	Ensemble of Models 1 & 3	0.911 (0.887–0.934)	0.494	0.662	0.832 (0.798–0.862)	0.829	0.834
7	Ensemble of Models 1 & 4	**0.921 (0.899–0.943)**	0.488	0.702	0.851 (0.819–0.880)	0.850	0.853
**Testing Dataset**							
1	Perinatal features only	0.841 (0.774–0.908)	0.550^a^	0.507^a^	0.752 (0.671–0.822) ^a^	0.810^a^	0.709^a^
5	Ensemble of Models 1 & 2	0.867 (0.806–0.928)	0.521^a^	0.576^a^	0.788 (0.710–0.853) ^a^	0.789^a^	0.788^a^
6	Ensemble of Models 1 & 3	0.884 (0.827–0.940)	0.494^a^	0.622^a^	0.810 (0.734–0.872) ^a^	0.844^a^	0.781^a^
7	Ensemble of Models 1 & 4	**0.899 (0.848–0.949)**	0.488^a^	0.623^a^	0.810 (0.734–0.872) ^a^	0.855^a^	0.773^a^

*DOL* Day of life, *ROC AUC* receiver operating characteristics area under the curve, *CI* confidence interval, *PPV* positive predictive value, *NPV* negative predictive value

^a^Based on the cutoff threshold generated using the training dataset

**Fig. 2:**
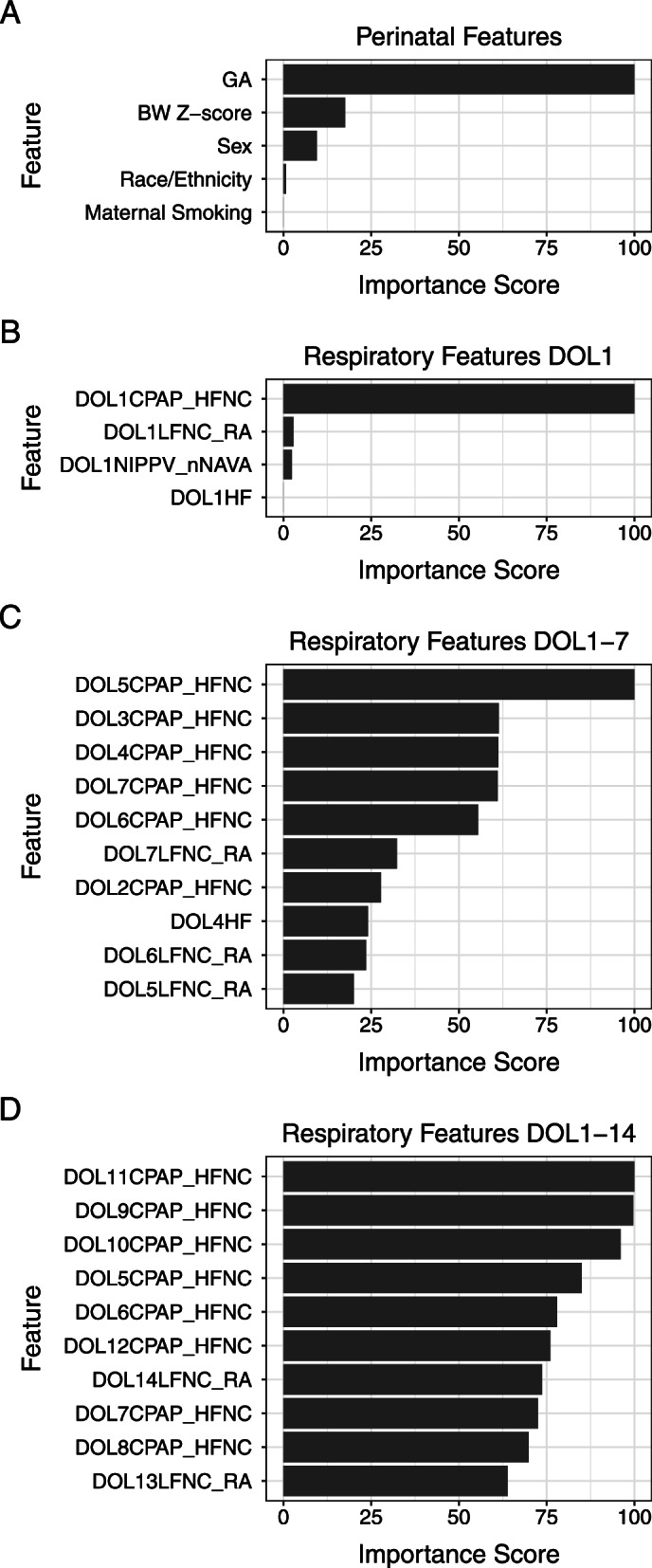
Feature importance scores for **A** Model 1 – five perinatal features, **B** Model 2—respiratory model data for day of life 1, **C** Model 3 – respiratory model data for day of life 1–7, and **D** Model 4 – respiratory model data for day of life 1–14. Feature importance scores were calculated based on permuting the values of the indicated feature followed by re-building the model and calculating the decrease in prediction accuracy. The scores were normalized between 0 and 100, with 0 being least important, and 100 being most important. The scores were obtained by running the varImp() function from the *caret* package

Using permutation to assess the relative importance of each feature, we found that gestational age was the most influential among all perinatal features, followed by birth weight z-score, male sex, maternal smoking, and race/ethnicity in the order of importance (Fig. 2A). For respiratory feature, CPAP/HFNC use was the most predictive of the outcome (Fig. 2B-D).

**Fig. 3:**
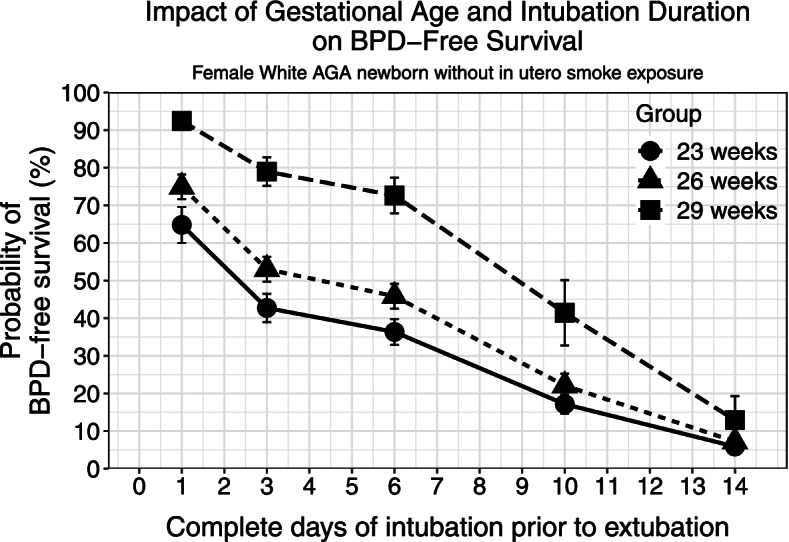
BPD-free survival probabilities of female, appropriate for gestational age, white, antenatally non-smoking exposed infants born at 23, 26, and 29 weeks of gestation intubated at birth for indicated periods. The error bars indicate standard errors of the probabilities. This plot depicts the simulated results from Scenario 1 (see text)

It is well established that lower gestational age at birth and longer intubation time are associated with higher risk of BPD. To confirm these facts in our model, Model 7 (perinatal features and all 14 days of respiratory data) was used to predict the probability of BPD-free survival (Scenario 1). As shown in Fig. 3, lower birth gestation and longer intubation time were individually associated with a reduced probability of BPD-free survival, providing assurance for the validity of the model.

**Fig. 4:**
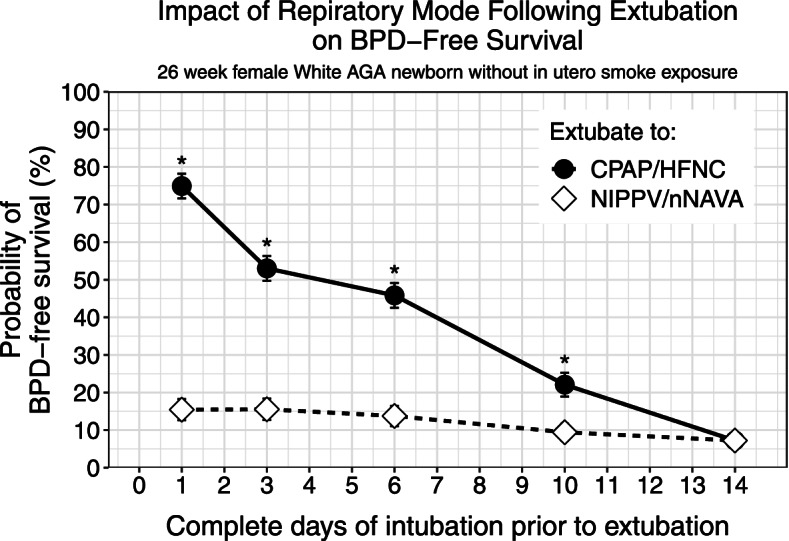
BPD-free survival probabilities of female, appropriate for gestational age, white, antenatally non-smoking exposed infants born at 26 weeks of gestation intubated at birth for the indicated periods followed by extubating to either continuous positive airway pressure (CPAP)/high-flow nasal cannula (HFNC) or to non-invasive positive pressure ventilation (NIPPV)/non-invasive neurally adjusted ventilatory assist (nNAVA). The error bars indicate standard errors of the probabilities. This plot depicts the simulated results from Scenario 2 (see text)

The model continued to be utilized for further clinical simulations. In scenario 2, we found that extubating to NIMV did not lead to a better respiratory prognosis, noting that the probability of BPD-free survival remained less than 20% even after only one day of intubation following birth, and that no statistically significant difference was shown between extubation and no extubation. On the other hand, if an infant were able to maintain adequate respiration on CPAP after extubation, the probability of BPD-free survival was significantly higher, although the probability decreased with more time spent intubated (Fig. 4).

**Fig. 5:**
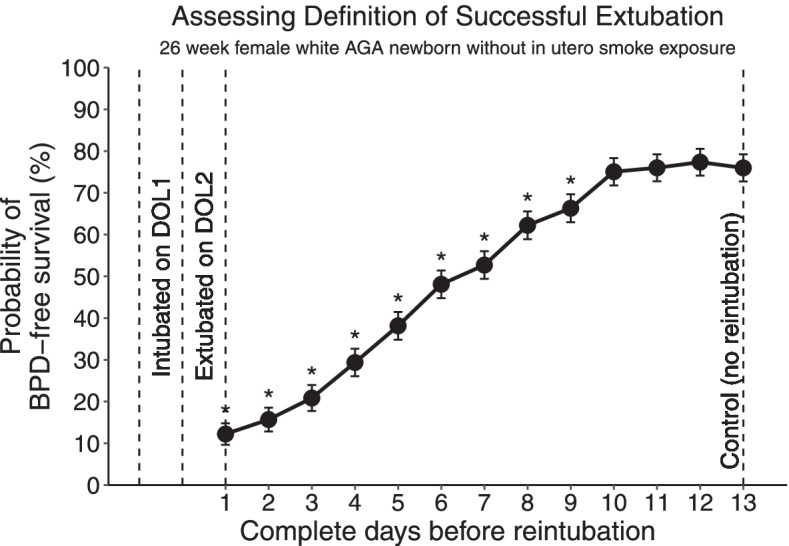
BPD-free survival probabilities of female, appropriate for gestational age, white, antenatally non-smoking exposed infants born at 26 weeks of gestation intubated at birth for one full day, followed by extubation between day of life 1 and 2, and reintubation following the indicated periods of time. In the control infant, there was no reintubation. Statistical comparison of the probability of BPD-free survival was made between the control infant and each of the infants who were reintubated individually using Student’s *t*-test. The asterisk sign (*) indicates *p*-value < 0.05. The error bars indicate standard errors of the probabilities. This plot depicts the simulated results from Scenario 3 (see text)

In Scenario 3, we observed that the longer an infant remained extubated before reintubation, the higher the probability of BPD-free survival (Fig. 5). After staying extubated for 9 complete days, there was no statistically significant difference in the probabilities of BPD-free survival between the reintubated infant and the non-reintubated (control) infant.

## Discussion

The literature suggests a developmental origin of BPD [13, 17, 18, 24, 26, 39–41]. In this study, we expanded on this concept to develop a machine learning BPD-free survival prediction model. Five non-modifiable perinatal factors combined with respiratory support mode data from the first 14 days of life were used in this model. The five perinatal factors selected for inclusion are based on the elegant work of Morrow et al. where biological, social, and antenatal exposure determinants were assessed for their association with BPD. While sex and race/ethnicity are strictly non-modifiable, the impact of maternal smoking, fetal weight gain, and birth gestation are factors that may be modifiable from the obstetric standpoint. Together, these factors indicate that low lung tissue volume at birth plays a determinant role in the development of BPD. We did not include maternal conditions (chorioamnionitis, preeclampsia, prolonged preterm rupture of membrane, etc.) or maternal medications (antenatal steroid, magnesium sulfate, antibiotics, etc.) due to inconsistent reliability of the information. Moreover, responses to these exposures were expected to be reflected on the trajectory of respiratory support mode use, including the reversibility of their influences.

For respiratory data, we chose to only include respiratory support mode data instead of other respiratory parameters such as the fraction of inspired oxygen, oxygen saturation, mean airway pressure, blood gas results, etc., because the respiratory support mode is the most reliable variable guided by physician orders and is less likely to be influenced by clinical subjectivity. Additionally, documentation of respiratory mode change is less likely to be associated with errors. Moreover, respiratory parameters with normal ranges or goal ranges are not useful by themselves because there would not be a clear distinction between the two outcomes groups to guide prediction. On the other hand, the decision to choose a respiratory support mode over another is heavily influenced by local respiratory protocols. Consequently, a model developed with local data may not be as easily generalizable. It is important to note that the study was not designed to assess the superiority of one specific respiratory mode over another within the same group.

The most well-developed and widely used prediction tool for BPD is the NICHD Neonatal Bronchopulmonary Dysplasia Outcome Estimator [27]. The model was developed with data from 2,415 infants that underwent internal and external validation with additional 1,214 and 722 infants, respectively, during development [26]. The model was further validated by two additional study cohorts (PreVILIG and PREMILOC), both showing fair discrimination [28, 29]. We and others showed that the Estimator performed well in predicting death or severe BPD [30, 31]. Unfortunately, the Estimator does have limitations as it does not have an option for infants of Asian descent nor an option for NIMV. One model was developed for each DOL which does not take into consideration time as a continuous factor. In their assessment, demographic factors play a more crucial role during the early days of life, which they justified based on a sequential addition of variables and the related ROC AUC values. Notably, this method has trouble assessing the contribution of respiratory trajectories to BPD risks across days of life because different models were built for each DOL.

In our design, we separated perinatal factors and respiratory data into two models during initial training. We combined the two models into a final ensemble model to ensure both perinatal and respiratory features were taken into consideration with equal weight. By taking this approach, the final model does not add more weight on the respiratory data for prediction. From the clinical standpoint, this approach not only provided us with an opportunity to assess the developmental origin of bronchopulmonary dysplasia, but will also allow us to assess BPD risks at four time points: at birth, at the end of the first 24 h, as well as at 7 and 14 complete days of life. The most appropriate model for risk stratification can be picked for use in future studies comparing efficacy of interventional therapies to prevent BPD development depending on the timing of the intervention. Although there is not an easily accessible and suitable algorithm in classic machine learning which considers repeated measurement in a way that is reminiscent of mixed-modeling, we felt that using a decision tree-based algorithm on a dataset with each DOL as one feature allows the model to assess the interrelationship of respiratory support modes across all days of life for each infant. This serves as an alternative approach to assessing changes in respiratory support mode over time.

The lack of improvement in BPD-free survival in infants extubated to a NIMV mode was unexpected (Scenario 2). Notably, more than 80% of the infants receiving NIMV received unsynchronized NIPPV instead of non-invasive NAVA. A multi-center prospective trial comparing NIPPV and CPAP use in infants born < 1,000 g (about 2.2 lbs), and < 30 weeks’ gestation showed no difference in the risks of BPD-free survival [42]. A subgroup analysis showed no difference in BPD risks among infants with prior intubation. The use of NIPPV did not appear to provide benefit for preventing subsequent reintubation which occurred in about 60% of the infants in both groups. The severity of illness from morbidities or comorbidities of prematurity did not differ between the two groups. These findings were different from other trials which assessed the early use of NIPPV compared to CPAP in infants born at a later gestational age, which showed reduced intubation needs and a lower risk of BPD [43–45]. A Cochrane review also found no difference in BPD risks between NIPPV and CPAP use [46]. In the input data for model training, we observed a higher percentage of infants without BPD who received CPAP/HFNC rather than NIMV (the majority received NIPPV) or an invasive ventilatory mode. Clinically, sicker infants were more likely to require longer NIMV support due to unreliable respiratory drives in order to avoid intubation/reintubation. Our model reflected that, which was different from the above-mentioned randomized controlled trial. Interestingly, a recent meta-analysis on mixed treatment comparisons found that surfactant administration followed by CPAP use provided superior respiratory outcomes to NIPPV use [47]. In clinical practice, it would be reasonable to trial CPAP with early use of pharmacological intervention to prevent apnea of prematurity on infants who are able to sustain a reliable respiratory drive, and reserve NIPPV to those infants who require artificial breath.

Prolonged intubation may be associated with barotrauma and volutrauma, while invasive ventilation may be necessary in poor pulmonary compliance or intolerance of non-invasive support. In the literature, extubation failure in preterm infants was inconsistently defined as reintubation between 48 h and 7 days [48–51]. Reintubation may be associated with respiratory setback, leaving us with the question of whether the interval between first extubation and reintubation conveys any significance in influencing long-term respiratory outcomes. Our prediction model provided an objective way of assessing what constitutes as the best definition for “successful extubation” (Scenario 3). We explored this by comparing the probability of BPD-free survival to a control simulated infant who did not require reintubation after initial extubation. Based on our simulation using BPD-free survival probability as outcome correlation, the duration required to maintain extubated is at least 48 h longer than the definitions used in most studies.

One major drawback of our model is that the source of the data was from infants cared for by the same group of neonatologists, so the study was considered as a single-center study, making external application of the model difficult. Nonetheless, studies have already suggested a strong influence of center effect on BPD rate and predictability [29, 32]. Plus, the goal of a prediction model is different from that of a statistical model. A prediction tool may be considered useful if it provides a reasonable balance between bias and variance, and is generalizable to a target population (e.g., preterm infants receiving care in one NICU or by one group of neonatal providers). Additional limitation of our prediction model was binary prediction of BPD-free survival, rather than prediction of BPD grade or severity. Also, the definition of BPD remained operational and subjective, affecting the accuracy of outcome labeling.

## Conclusions

In conclusion, we developed a prediction tool for BPD-free survival based on the developmental origin of BPD. The predictability of BPD using early-life predictors provides an opportunity for personalized early intervention targeting only the at-risk subpopulation to minimize potential harm to the low-risk group. Moreover, the ability to simulate respiratory support use provides clinicians an objective way of assessment for an optimal respiratory support mode to endow actual benefit to the infant. A web app created to demonstrate the use of the prediction tool developed in this study is accessible at https://neostat.shinyapps.io/Resp_Mode_ML/.

## Data Availability

The datasets used and analyzed during the current study are available from the corresponding author on reasonable request.

## Abbreviations

BPD: Bronchopulmonary dysplasia
PMA: Postmenstrual age
IUGR: Intrauterine growth restriction
NICU: Neonatal intensive care unit
HFV: High-frequency ventilation
CMV: Conventional mechanical ventilation
NIMV: Non-invasive mechanical ventilation
NIPPV: Non-invasive positive pressure ventilation
NAVA: Neurally adjusted ventilatory assist
CPAP: Continuous positive airway pressure
HFNC: High-flow nasal cannula
LFNC: Low-flow nasal cannula
DOL: Day of life
ROC AUC: Receiver operating characteristic area under the curve
